# Reasons for and Congruence Between Preferred and Actual Place of Death Among Cancer Patients Receiving End-of-Life Care: A Cross-Cultural Multicenter Prospective Cohort Study in East Asia

**DOI:** 10.3390/cancers17132062

**Published:** 2025-06-20

**Authors:** Chiu-Hsien Yang, Chien-Yi Wu, Shao-Yi Cheng, Masanori Mori, Sang-Yeon Suh, Sun-Hyun Kim, Wen-Yuan Lin, Takashi Yamaguchi, Hsien-Liang Huang, Jun Hamano, Yusuke Hiratsuka, Satoru Tsuneto, Tatsuya Morita, Ping-Jen Chen, on behalf of the EASED Investigators

**Affiliations:** 1 Department of Family Medicine, Kaohsiung Medical University Hospital, Kaohsiung Medical University, Kaohsiung 807378, Taiwan; 970434@kmuh.org.tw (C.-H.Y.); chienyi@kmu.edu.tw (C.-Y.W.); 2 School of Medicine, College of Medicine, Kaohsiung Medical University, Kaohsiung 807378, Taiwan; 3 Department of Family Medicine, College of Medicine and Hospital, National Taiwan University, Taipei 10051, Taiwan; scheng2140@gmail.com (S.-Y.C.); solomonhuang@ntu.edu.tw (H.-L.H.); 4 Division of Palliative and Supportive Care, Seirei Mikatahara General Hospital, Hamamatsu 433-8558, Japan; masanori.mori@sis.seirei.or.jp (M.M.); tmorita@sis.seirei.or.jp (T.M.); 5 Department of Family Medicine, Dongguk University Ilsan Hospital, Goyang-si 10326, Republic of Korea; lisasuhmd@hotmail.com; 6 Department of Medicine, College of Medicine, Dongguk University, Seoul 04620, Republic of Korea; 7 Department of Family Medicine, School of Medicine, Catholic Kwandong University, International St. Mary’s Hospital, Incheon 22711, Republic of Korea; sunhyun@ish.or.kr; 8 Department of Family Medicine, China Medical University Hospital, China Medical University, Taichung 40447, Taiwan; 9 Division of Palliative Medicine, Kobe University Graduate School of Medicine, Kobe 650-0017, Japan; ikagoro@pop06.odn.ne.jp; 10 Division of Clinical Medicine, Faculty of Medicine, University of Tsukuba, Tsukuba 305-8577, Japan; junhamano@md.tsukuba.ac.jp; 11 Department of Palliative Medicine, Tohoku University School of Medicine, Sendai 980-8575, Japan; hiratsuka.med.t@gmail.com; 12 Department of Human Health Sciences, Graduate School of Medicine, Kyoto University, Kyoto 606-8501, Japan; tsuneto@kuhp.kyoto-u.ac.jp; 13 National Center for Geriatrics and Welfare Research, National Health Research Institutes, Miaoli 350401, Taiwan; 14 School of Medicine, College of Medicine, National Sun Yat-sen University, Kaohsiung 80424, Taiwan

**Keywords:** cancer care, congruence, cross-cultural, end-of-life care, palliative care, place of death

## Abstract

**Background/Objectives**: The place where a person dies serves as an indicator of end-of-life care quality. This study aims to identify the congruence of place of death (POD) and the reasons behind decision-making among terminally ill cancer patients in East Asia. **Methods**: We conducted a prospective multicenter cohort study in palliative care units in Japan, Korea, and Taiwan. Data were collected by the responsible physicians during routine clinical practice. Sankey diagrams were applied to present changes in reasons for POD incongruence. **Results**: A total of 2638 participants from 37 palliative care units in the three countries were enrolled, and most of them died at PCUs (Japan: 95.7%, Korea: 94%, Taiwan: 82%, *p* < 0.05). The congruence rate of the preferred and actual POD among PCU inpatients ranged from 70% to 80%. Availability of end-of-life care is the most common reason for preferred and eventual PCU death (78.6%, 72.2%, and 52.1%, respectively, *p* < 0.05). However, 13–22% of patients admitted to PCUs still preferred to die at home, for which traditional culture (20.2%, 40.8%, and 82%, respectively, *p* < 0.05) or family influence (44.4%, 38.8%, and 51.7%, respectively, *p* > 0.05) are the main reasons. Cultural and environmental factors, such as influences from family members’ preferences in the three countries or the ownership of housing/housing settings in Japan, are the main challenges in achieving POD congruence. **Conclusions**: Culturally inclusive strategies in clinical practice and policy implementation for identifying the preferred POD, enhancing communication among stakeholders, and facilitating transitional support may improve the quality of goal-concordant care.

## Simple Summary

Place of death reflects not only personal choice but also the quality and accessibility of care, as well as culturally specific factors like family-centered decision-making in East Asia. This study followed over 2600 terminally ill cancer patients admitted to palliative care units in Japan, Korea, and Taiwan. We found that while 13–22% of these patients still preferred to die at home, the majority (82–96%) ultimately died in hospitals or hospices. Even within specialized palliative care, the congruence between the preferred and actual place of death was high (70–80%) only for institutional deaths, while home death rarely occurred. Family opinions, cultural traditions, and housing situations often play a strong role in shaping these outcomes. This first large cross-cultural cohort study deepens our understanding of clinical- and system-level barriers to goal-concordant care and provides actionable insights for improving communication, transitional planning, and community support in East Asian healthcare systems.

## 1. Introduction

Place of death (POD) is a quality indicator of end-of-life care [1] and has an impact on the quality of dying and death for patients with life-limiting illness [2,3]. Dying in one’s preferred place or a place familiar to one is related to a good death [4]. International investigations on this topic have mainly been conducted in Europe and the United States [5,6,7]. In East Asia, previous studies revealed that home death preference ranged from 40 to 65% [8,9,10,11]; nevertheless, the proportion of hospital deaths continues to account for three-quarters of deaths [1], and the place of end-of-life care and POD remain ethical dilemmas of priority in East Asian countries [12]. The gap regarding death in preferred places underscores the need for a cross-cultural comparison of POD in areas with a rapidly aging population.

Patients’ preference for life-sustaining treatments and POD may change over time or when patients experience actual functional decline or are reaching the end of life [13,14,15,16]. Study participants with different disease severities or in different care settings in studies for POD issues may present remarkably different results and meanings. The study of POD in Asia, which emphasizes patients’ preferences at the very end of life to understand their last wishes, is less investigated.

The congruence of the preferred and actual POD represents the realization of patient autonomy in end-of-life decisions and the quality of patient-centered care [15,17]. Differences in geography, culture, and healthcare systems across countries may exert a decisive influence on POD and the likelihood of achieving death in the preferred POD [7,18,19]. A recent systematic review reported that the current evidence regarding POD congruence has mainly been based on retrospective observational design, and congruence values varied substantially across countries and populations [20]. The POD congruence ranged between 41% and 72% in high-income countries in Europe and America [20,21,22,23,24,25], but studies in Asia are scarce.

Large prospective cross-cultural studies investigating POD congruence and the reasons behind POD-related decisions using a unified questionnaire have not been conducted in Asian countries. Hence, we aimed to conduct an international multicenter study in three East-Asian countries and identify the POD congruence and its reasons for decision-making among terminally ill cancer patients who were admitted to PCUs.

## 2. Materials and Methods

We conducted a nationwide, prospective cohort study in multiple PCUs across Japan, Korea, and Taiwan, specifically known as the East-Asian collaborative cross-cultural Study to Elucidate the Dying Process (EASED). In Japan, informed consent was waived because of the observational nature of this study and in accordance with the ethical guidelines for human research of Japan’s Ministry of Health, Labor, and Welfare. In Korea and Taiwan, informed consent was obtained from the patients or their families in cases where the patient was not capable of making decisions. This study was approved by the institutional review board of Seirei Mikatahara General Hospital (Research No. 16–29) and by all participating institutions in each country. We registered the study at UMIN-CTR (UMIN00002545; URL: https://center6.umin.ac.jp/cgi-open-bin/ctr_e/ctr_view.cgi?recptno=R000029294, accessed on 6 June 2025).

### 2.1. Participants

New inpatients consecutively admitted to the participating PCUs during the study period were enrolled. All observations were performed during routine clinical practice. The inclusion criteria for patients were (1) adult (≥18 years in Japan and Korea and ≥20 years in Taiwan), (2) having locally extensive or metastatic cancer, and (3) admitted to participating PCUs. The exclusion criteria were (1) scheduled discharge within 1 week and (2) refusal of patients or their families to be enrolled.

### 2.2. Data Collection

The primarily responsible palliative care physicians evaluated their patients in PCUs and recorded the information using standardized forms during routine clinical practice. We collected patients’ demographic data, including age, sex, primary cancer site, education level, marital status, religion, and Eastern Cooperative Oncology Group Performance Status at admission. Preferred POD and its reasons were recorded at PCU admission. Actual POD was documented by PCU team staff through routine process when death was confirmed during follow-up. Options of decisional reasons for preferred and actual POD were summarized according to the literature review, findings from our previous study, and discussion in investigators’ panel meetings in the three countries [26]. We collected data from January 2017 to September 2018.

### 2.3. Data Analysis

Descriptive statistics were used to represent patients’ demographic characteristics, POD preference, actual POD, and the reasons for POD decision. We categorized the POD into two groups when presenting the reasons: “Institutional death” included death at PCU/hospice, general ward or emergency room. “Home death” referred to death at the patient’s or relatives’ home, or care/nursing homes. Care home is referred to long-term residential facilities providing non-hospital-based custodial or specific care for diseases and conditions, including nursing homes. The congruence between preferred and actual POD was calculated and presented as a percentage. We performed chi-square analysis to examine the differences for categorical variables, analysis of variance (ANOVA) tests for the continuous variable across three countries, and defined *p* values < 0.05 as significantly different. Post hoc comparisons were conducted using pairwise chi-squared tests with Bonferroni correction to identify specific country differences in the reasons for POD decision. We applied Sankey diagrams to present changes in reasons for POD incongruence. All analyses were performed using MedCalc Statistical Software version 18.11.6 (MedCalc Software, Ostend, Belgium) and VP Online Drawing Tool (Sankey diagram).

## 3. Results

### 3.1. Patient Characteristics

**Figure 1:**
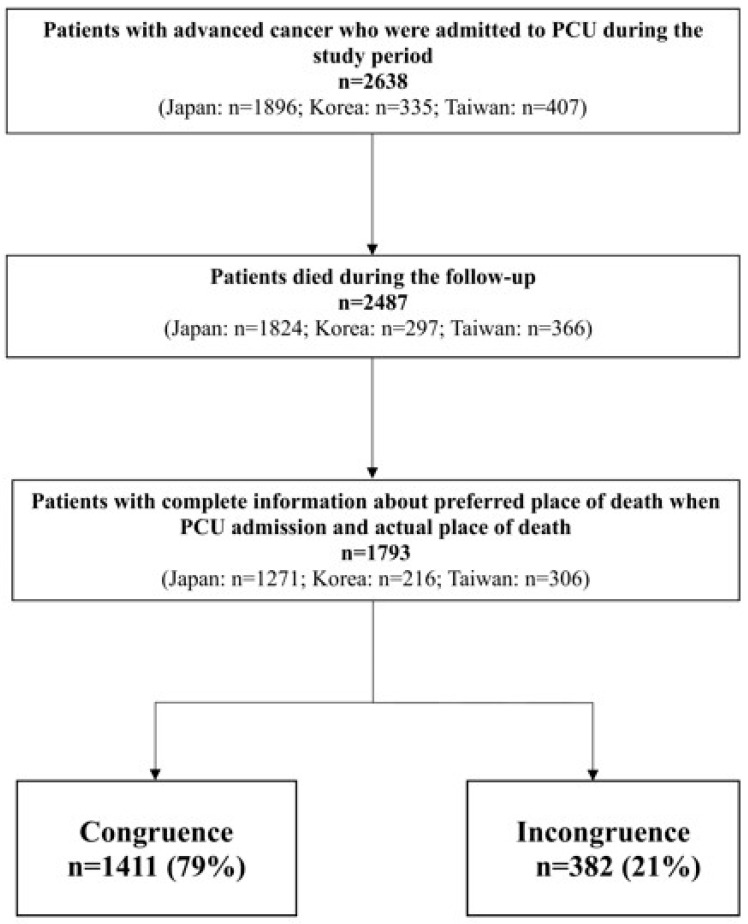
Flowchart of case selection.

A total of 2685 patients were initially enrolled from 37 palliative care units between January 2017 to September 2018, including 22 units in Japan, 11 in South Korea, and 4 in Taiwan. After excluding patients lost to follow-up, 2638 participants were included in our analysis (Figure 1). Table 1 presents the demographic characteristics of patients across the three countries. Overall, 52% were male (n = 1375) and the mean age of the participants was 72.4 ± 12.3 years in Japan, 68.1 ± 12.2 years in Korea, and 66.0 ± 13.8 years in Taiwan. The length of PCU stay (days) was 26.9 in Japan, 25.9 in Korea, and 14.3 in Taiwan. Additional characteristics of the patients are summarized in Table 1.

### 3.2. Preferred and Actual POD

During the follow-up, 2487 patients died, and Table 2 shows the preferred POD of 2638 participants and actual POD of decedents across the three countries. Table 2 presents the preferred and actual POD in the three countries. In Taiwan, a higher proportion of patients expressed their preferred POD, 245 (60.2%) for PCU/hospice, 88 (21.6%) for home, which were higher than the percentages in Japan and Korea (*p* < 0.05). Notably, a high percentage of unknown or no preference of POD was observed, especially in Japan and Korea (30.1% and 26%, respectively). At the end of follow-up, the majority of patients died in a PCU/hospice: up to 95% in Japan and Korea, and 82% in Taiwan. In Taiwan, 13.7% of patients died at home.

### 3.3. Reasons for and Congruence of POD

**Figure 2:**
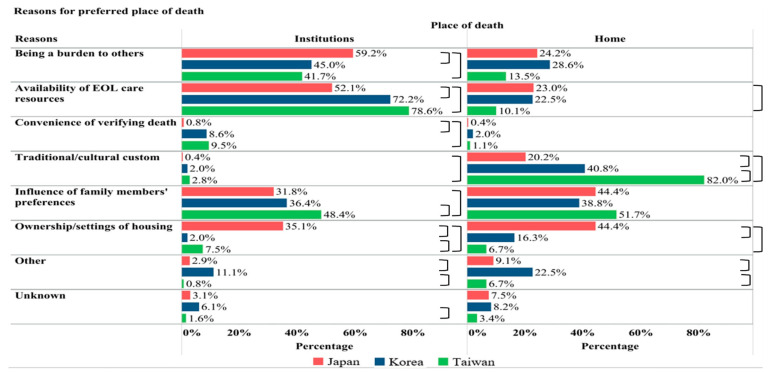
Reasons for preferred place of death (n = 1911). EOL: end-of-life. 〕= significantly different (*p* < 0.05) between two countries. Institution deaths included PCU/hospice/general ward/ER; home death included patients’ or relatives’ homes or care homes. Reasons for patients who has other (n = 5) or unknown/no (n = 722) preferred place of death are not included. Multiple reasons can be chosen.

Reasons for preferred POD are shown in Figure 2. Among patients who preferred hospital death, the most commonly reported reasons in Japan and Korea were “availability of end-of-life care resources” and “being a burden to others”. In contrast, in Taiwan, the primary reason was the availability of resources, followed by “influence of family members’ preferences”.

Among patients who preferred home death, in Korea and Taiwan, the most common reason was “traditional/cultural custom” (82% and 41%, respectively), followed by “influence of family members’ preferences”. In contrast, in Japan, “ownership/settings of housing” and “influence of family members’ preferences” were equally the most frequently reported reasons.

**Figure 3:**
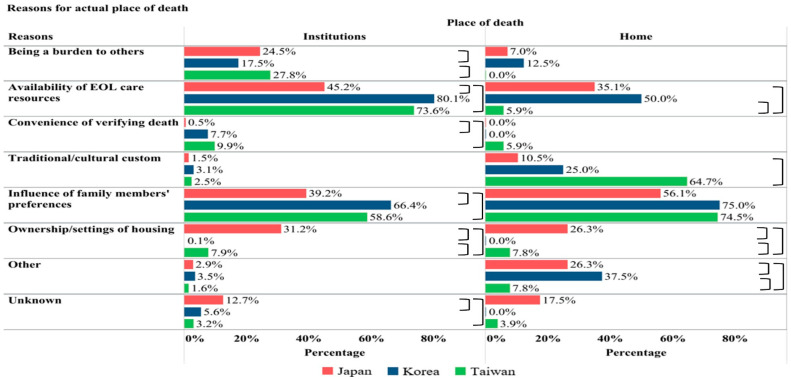
Reasons for actual place of death (n = 2482). EOL: end-of-life. 〕= significantly different (*p* < 0.05) between two countries. Institution deaths included PCU/hospice/general ward/ER; home death included patients’ or relatives’ homes or care homes. Five patients with missing data on place of death were excluded. Multiple reasons can be chosen.

Figure 3 illustrates reasons for actual POD. Among patients who eventually died in the hospital, the two most common reasons were the same in the three countries: “availability of end-of-life care resources” and “influence of family members’ preferences”. These two reasons accounted for the overwhelming majority in Korea (80% and 66.4%).

Among patients who died at home or care homes, the most commonly reported reason across all three countries was “influence of family members’ preferences”. In Japan and Korea, the second most common reason was “availability of end-of-life care resources”, whereas this only accounted for 6% of cases in Taiwan. In Taiwan, the second leading reason was “traditional/cultural custom”. Notably, “ownership/settings of housing” remained the third common reason for both institutional and home deaths in Japan.

Patients with both a documented preference for POD and information on actual POD (n = 1793) were included in the analysis of POD congruence (Figure 1). The percentage rate of overall POD congruence among cancer patients receiving end-of-life care ranged from the highest rate in Japan (80.2%; 95% CI, 77.9–82.3%), followed by Taiwan (78.4%; 95% CI, 73.8–83.0%) and the lowest rate in Korea (70.4%; 95% CI, 64.2–76.6%) (Table S1). In contrast to the relatively high congruence of institutional death, the congruence rates for home death were markedly low across all three countries (Japan: 10.7%, Korea: 6.1%, Taiwan: 39.8%; *p* < 0.05). The most frequent pattern of incongruence involved patients who initially preferred to die at home but ultimately died in a PCU or hospice.

**Figure 4:**
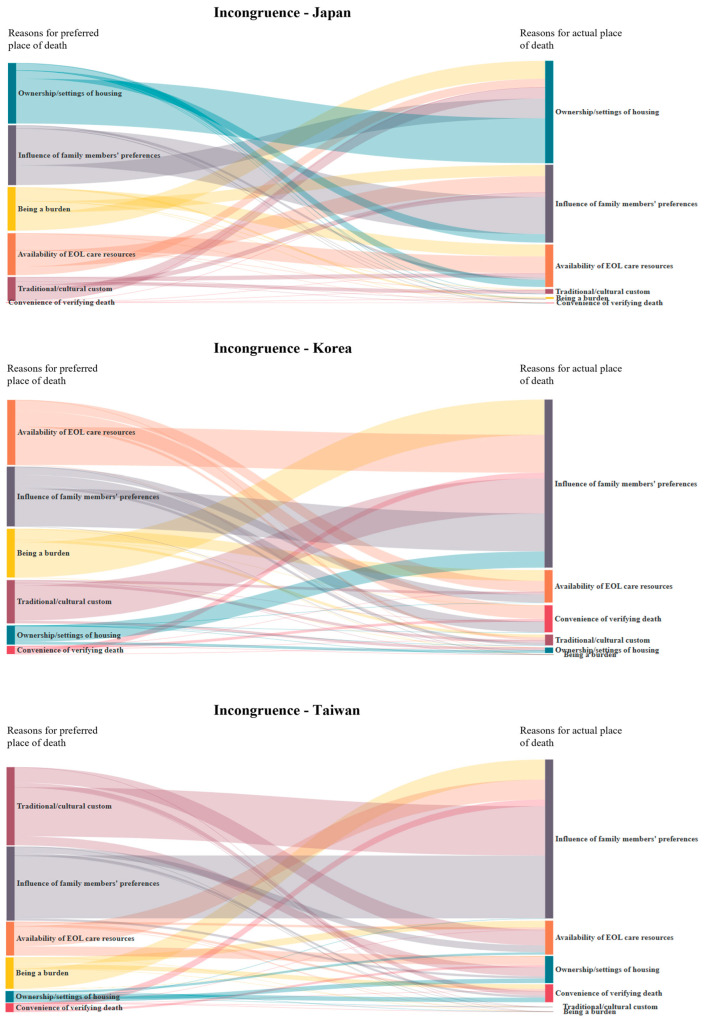
Change in the reasons of incongruence between preferred and actual POD among terminally ill cancer patients.

Figure 4 illustrates Sankey diagrams regarding changes in reasons for POD incongruence in the three countries. In Japan, “ownership/setting of housing” was the decisive reason for the POD incongruence group. In contrast, “influence of family members” played the most critical role in POD incongruence in Taiwan and Korea.

## 4. Discussion

This is the largest prospective cohort study conducted in East Asian countries to compare the congruence of preferred and actual POD, as well as identifying the underlying reasons for POD-related decision-making among terminally ill cancer patients. The POD congruence rate among PCU inpatients was 70–80% in Japan, Korea, and Taiwan. Our findings highlight that cultural and environmental factors, such as influences of family members’ preferences across all three countries, or ownership/settings of housing in Japan, are the main gaps for POD congruence. Notably, 13–22% of patients admitted to PCUs as the place of care still preferred to die at home due to traditional culture or families’ influence; however, most of them ultimately died in PCUs. Our findings suggest the need for the timely identification of patients’ preferred POD, improved communication with family members, and appropriate transitional support to achieve better goal-concordant care within specific cultural contexts.

### 4.1. POD Congruence and Its Reasons

Determinants such as disease type, length of disease trajectory, symptom control, and individual demographic factors may influence POD congruence [23,27,28]. An increasing body of literature has shown that discussion with healthcare providers regarding POD, caregivers’ stress and preferences, and the use of home care services [6,29,30], as well as socioeconomic and environmental factors, such as social support, geographic distance and place of residence also have a substantial impact on achieving POD congruence [20,31,32]. In our study, the primary pattern of incongruence between preferred and actual POD was that patients initially preferred to die at home but eventually died in PCU/hospice. The influences of family members’ preferences emerged as the predominant factor contributing to POD incongruence in all three countries, especially in Korea and Taiwan. These results may be explained by the family-centered decision-making style in Confucian-influenced society, where patients’ autonomy of end-of-life care is often diminished or overridden by family preferences [33,34].

In Japan, the major barrier to achieving POD congruence is related to housing ownership and settings. This may be attributed to high population density in urban areas, the rise in nuclear family structures, an increasing proportion of individuals not living with family, a shortage of home-based caregivers, and the socio-economic decline following the 1990s. Recent government-led housing policies, such as Elderly Housing with Care Services in collaboration with the Home-Visit Nursing Agencies, may enhance the capacity to support patients’ preference for home death [35]. These efforts also reflect the unmet needs for coordinated transitional care and adequate home-based end-of-life care to address the low POD congruence among patients who preferred to die at home in the three countries. In Taiwan, most home-based palliative care is delivered by interdisciplinary palliative care specialists from hospitals who provide outreach services to patients in the community. In contrast, end of life care in Japan is primarily provided by non-specialist primary care physicians, while in Korea, palliative care nurses constitute the main providers [26]. The specialist-led model of home-based care in Taiwan may contribute to higher rates of POD congruence compared to those in Japan and Korea [30,36].

### 4.2. Reasons for Preferred and Actual POD

The availability of end-of-life care resources was the most commonly reported reason for both the preference for and the occurrence for hospital death across the three countries. Being a burden to their family constituted another important reason for choosing death in hospitals, especially in Japan. These findings are observed not only in Asian culture but also in European countries [28,37], where providing care and ensuring patient comfort often take precedence over achieving the preferred POD. When the care demand at the end-of-life stage becomes overwhelming, family caregivers may feel unable to cope with the stress of the patient dying at home, leading them to reconsider or withdraw their support for home death [38]. On the other hand, the occurrence of home death is associated with the availability of care at home when living with relatives (expanded family support) and the preference of caregiver [27]. The rise in nuclear families and the increasing number of married women in the workforce have contributed to a shortage of available caregivers. These factors may help explain why the influence of family members has become increasingly critical in determining the actual POD.

In Taiwan and Korea, up to 82% and 40.8% of preferences for home deaths, respectively, were influenced by traditional and cultural customs. In Taiwan, one such belief is captured by the idiom “luò yè guī gēn” (fallen leaves will return to the roots) which reflects the deep cultural value placed on dying at home and may strongly affect patients’ end-of-life decision [39]. In Korea, a similar cultural belief holds that dying away from home, known as “gaeksa” is considered a bad death. This phenomenon was also found in our previous international comparison study [26], in which Taiwanese patients and their families expressed a preference for home death home based on the belief that the patients’ soul may not be able to return from the hospital if they die in hospital.

### 4.3. Preferred POD at Different Stages in Disease Trajectory

Lack of awareness regarding the need to consider or express a preference for POD was often observed in our study and previous evidence [15]. When patients and their family caregivers are informed of the incurable or terminal nature of illness, they may be more likely to engage in the discussion about POD preferences with healthcare professionals, which in turn can increase the likelihood of home death or improve POD congruence [15,29,40]. In Taiwan, inpatients and their families in PCUs were informed of the patients’ impending death at a much higher rate and at an earlier stage compared to their counterparts in Japan and Korea (66.4%, 4.8%, and 19.6%, respectively), which may partially explain the lower proportion of unknown or no preference for POD in Taiwan [41].

Patients may revise their treatment goals and end-of-life care preferences as their functional status declines [13,14,15]. They may also opt for hospital admission to achieve better symptom control rather than remain at home [22]. The relatively low proportion of patients who preferred home death (12–22%) in our study was remarkably lower than the rates reported in previous studies conducted in the three countries, and this difference may be attributed to variations in study populations. Our study samples consisted of terminally ill cancer inpatients in PCUs. In contrast, prior research focused on the general population, patients with earlier stages of cancer, or individuals with terminal illness who were not receiving hospice care [9,10,11].

### 4.4. Strengths and Limitations

The strengths of this study include its prospective design, multicenter setting, and large sample size across three countries. We conducted cross-cultural comparisons using structured questionnaires that were administered during the same period. In addition, a low rate of missing data related to death events or loss of follow-up enhances the reliability of our analysis. Third, culturally adapted reasons for POD preferences and congruence provide valuable insights into real-world circumstances for POD decision-making in terminally ill cancer patients, instead of scenario-based studies involving healthier populations. Nevertheless, several limitations should also be acknowledged. First, the study population consisted of terminal patients admitted to PCUs from various wards or home, which may limit the external validity of our findings. The distribution of preferred POD may have been skewed toward PCU settings, and therefore the results may not apply to terminally ill patients at home or in other community settings. Second, the mental capacity of patients to communicate their preferences and reasons may be impaired. Medical professionals may not clearly identify the information about POD if patients did not discuss this with their family. Third, patients’ POD preferences may have changed over time due to disease progression and the evolving dynamics between therapeutic alliance during PCU admission. Fourth, the relative high proportion of unknown or no preference in Japan and Korea may limit this interpretation.

## 5. Conclusions

We identified specific cultural and socio-economic reasons underpinning the POD decision-making in East Asia, including the influences of family preferences across the three countries, housing-related challenges in Japan, and traditional customs in Taiwan. Culturally inclusive strategies in clinical practice and policy implementation—such as the timely identification of the preferred POD during routine assessments, enhancing communication among stakeholders through family meetings, and facilitating transitional support via community or home care teams—may improve the quality of goal-concordant care. Future studies are warranted to explore how patients and bereaved families perceive POD congruence in different cultural contexts and its association with end-of-life care outcomes. Diverging preferences for home as the POD across different eras and regions highlight the need for further investigation. Cross-regional comparisons between Asia and other countries in Europe or North America would be highly valuable to explore the systemic influences on POD preferences and outcomes in diverse healthcare settings.

## Figures and Tables

**Table 1:** Demographic profiles of cancer patients admitted to palliative care units in three East-Asian countries (n = 2638).

	Japan n = 1896	Korea n = 335	Taiwan n = 407	*p* Value
Age ^a^ [years, mean ± SD]	72.4 ± 12.3	68.3 ± 12.2	66.6 ± 13.8	<0.001
Sex, n (%)				0.1295
Male	965 (50.9)	184 (54.9)	226 (55.5)	
Female	931 (49.1)	151 (45.1)	181 (44.5)	
Primary cancer site, n (%)				<0.001
Lung	319 (16.8)	49 (14.6)	77 (18.9)	
Gastroesophageal	265 (14.0)	45 (13.4)	28 (6.9)	
Colorectal	254 (13.4)	52 (15.5)	56 (13.8)	
Hepatobiliary/Pancreas	363 (19.1)	96 (28.7)	99 (24.3)	
Breast	131 (6.9)	19 (5.7)	18 (4.4)	
Gynecological	119 (6.3)	15 (4.5)	17 (4.2)	
Urological	141 (7.4)	16 (4.8)	27 (6.6)	
Head/Neck	68 (3.6)	8 (2.4)	48 (11.8)	
Others	236 (12.4)	35 (10.4)	37 (9.1)	
Highest level of education ^b^, n (%)				<0.001
<High school	58 (3.1)	157 (46.9)	224 (55.0)	
High school/Some college	184 (10)	118 (35.2)	133 (32.7)	
≥College degree	127 (6.7)	49 (14.6)	43 (10.6)	
Living with family ^c^, n (%)	1376 (72.8)	293 (87.5)	375 (92.1)	<0.001
Marital status ^d^, n (%)				<0.001
Married	1151 (60.7)	227 (67.8)	250 (61.4)	
Widowed	403 (21.3)	67 (20.0)	89 (21.9)	
Unmarried	205 (10.8)	13 (3.9)	29 (7.1)	
Separated	113 (6.0)	26 (7.8)	39 (9.6)	
Religion, n (%)				<0.001
No religion	822 (43.4)	121 (36.1)	60 (14.7)	
Buddhism and Taoism	206 (10.9)	75 (22.4)	225 (55.3)	
Christianity	38 (2.0)	133 (39.7)	24 (5.9)	
Others or unknown	830 (43.8)	6 (1.8)	98 (24.1)	
ECOG performance status, n (%)				<0.001
0–2	184 (9.7)	89 (26.6)	41 (10.1)	
3	797 (42.0)	158 (47.2)	123 (30.2)	
4	915 (48.3)	88 (26.3)	243 (59.7)	
Communication capacity at admission ^e^, n (%)				<0.001
0	980 (51.7)	175 (52.2)	156 (38.3)	
1	520 (27.4)	115 (34.3)	121 (29.7)	
2	211 (11.1)	20 (6.0)	58 (14.3)	
3	185 (9.8)	23 (6.9)	72 (17.7)	
Length of stay (days) ^f^	26.9	25.9	14.3	<0.001

ECOG: Eastern Cooperative Oncology Group; SD: standard deviation. ^a^ Missing number = 1 in Taiwan; ^b^ missing number = 1527 in Japan, missing number = 11 in Korea, missing number = 7 in Taiwan; ^c^ missing number = 22 in Japan, missing number = 1 in Korea; ^d^ missing number = 24 in Japan, missing number = 2 in Korea; ^e^ missing number = 2 in Korea; ^f^ missing number = 6 in Japan, missing number = 2 in Taiwan.

**Table 2:** Preferred place of death and actual place of death among the PCU patients in Japan, Korea, and Taiwan.

	Preferred Place of Death *			Actual Place of Death ^#^		
	Japan n = 1896	Korea n = 335	Taiwan n = 407	Japan n = 1824	Korea n = 297	Taiwan n = 366
**Place of death**, n (%)						
PCU/hospice	1045 (55.1)	170 (50.7)	245 (60.2)	1746 (95.7)	279 (94.0)	300 (82.0)
General ward	26 (1.37)	28 (8.4)	7 (1.7)	19 (1.0)	5 (1.7)	10 (2.7)
Own home	244 (12.9)	49 (14.6)	88 (21.6)	55 (3.0)	5 (1.7)	50 (13.7)
Care/nursing home	7 (0.4)	0 (0)	1 (0.3)	1 (0.1)	3 (1.0)	1 (0.3)
Home of a relative/friend	1 (0.1)	0 (0)	0 (0)	1 (0.1)	0 (0)	0 (0)
Other	3 (0.2)	1 (0.3)	1 (0.3)	1 (0.1)	2 (0.7)	4 (1.1)
Unknown/No preference	570 (30.1)	87 (26.0)	65 (16.0)	N/A	N/A	N/A

N/A: not applicable; PCU: palliative care unit. * Distribution of preferred place of death indicates significance *p* < 0.05 among three countries by χ^2^ tests. ^#^ Five patients with missing data on place of death are not included; distribution of actual place of death indicates significance *p* < 0.05 among three countries by χ^2^ tests.

## Data Availability

The datasets generated and/or analyzed during the current study are not publicly available due to institutional and ethical restrictions concerning patient privacy, but are available from the corresponding author on reasonable request.

## Supplementary Materials

The following supporting information can be downloaded at: https://www.mdpi.com/article/10.3390/cancers17132062/s1, Table S1: Congruence between preferred and actual place of death among terminally ill cancer patients admitted to PCUs (N = 1793).
