# Supplementary material for: Reasons for and Congruence Between Preferred and Actual Place of Death Among Cancer Patients Receiving End-of-Life Care: A Cross-Cultural Multicenter Prospective Cohort Study in East Asia
Source: Cancers (Basel). 2025 Jun 20;17(13):2062. doi: 10.3390/cancers17132062 (PMC12248815; doi:10.3390/cancers17132062)
Supplement: Supplementary file 1 [file cancers-17-02062-s001.zip › cancers-3657645-supplementary.pdf]

Supplementary materials

Table S1 Congruence between preferred and actual place of death among terminally ill cancer patients admitted to PCUs (N=1793)

	**Japan N =1,271**		**Korea N =216**		**Taiwan N =306**	
	n (%)	Rank	n (%)	Rank	n (%)	Rank
**Congruence**	1019 (80.2)		152 (70.4)		240 (78.4)	
PCU/hospice	991	1st	146	1st	205	1st
Own home	26	2nd	3	2nd	35	2nd
Others	2		3		0	
**Incongruence**	252 (19.8)		64 (29.6)		66 (21.6)	
Own home → PCU/hospice	196	1st	41	1st	38	1st
General ward → PCU/hospice	23	2nd	14	2nd	7	4rd
PCU/hospice → Own home	10	4rd	1	4th	8	3nd
Others	23		8		13	

PCU: palliative care unit.

1. Excluding patients who have unknown preferred place of death or unknown actual place of death (n=694).
